# Single and few cell analysis for correlative light microscopy, metabolomics, and targeted proteomics†

**DOI:** 10.1039/d4lc00269e

**Published:** 2024-08-08

**Authors:** Luca Rima, Christian Berchtold, Stefan Arnold, Andri Fränkl, Rosmarie Sütterlin, Gregor Dernick, Götz Schlotterbeck, Thomas Braun

**Affiliations:** a Biozentrum, University of Basel Spitalstrasse 41 Basel Switzerland; b Hochschule für Life Sciences, FHNW Fachhochschule Nordwestschweiz Switzerland; c F. Hoffmann-La Roche Ltd Switzerland

## Abstract

The interactions of proteins, membranes, nucleic acid, and metabolites shape a cell's phenotype. These interactions are stochastic, and each cell develops differently, making it difficult to synchronize cell populations. Consequently, studying biological processes at the single- or few-cell level is often necessary to avoid signal dilution below the detection limit or averaging over many cells. We have developed a method to study metabolites and proteins from a small number of or even a single adherent eukaryotic cell. Initially, cells are lysed by short electroporation and aspirated with a microcapillary under a fluorescent microscope. The lysate is placed on a carrier slide for further analysis using liquid-chromatography mass spectrometry (LC-MS) and/or reverse-phase protein (RPPA) approach. This method allows for a correlative measurement of (i) cellular structures and metabolites and (ii) cellular structures and proteins on the single-cell level. The correlative measurement of cellular structure by light-microscopy, metabolites by LC-MS, and targeted protein detection by RPPA was possible on the few-cell level. We discuss the method, potential applications, limitations, and future improvements.

## Abstract 2

We combined a single-cell lysis and handover system with mass spectrometry and reverse-phase protein arrays, allowing correlative single- and few-cell analysis combining microscopy with metabolomics and targeted proteomics.

## Graphical abstract

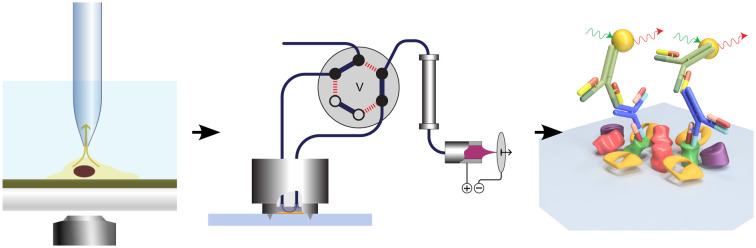

## 1 Introduction

A complex interplay of proteins, lipid membranes, nucleic acids, and metabolites defines the phenotype of biological systems. However, analyzing these interaction networks is complicated by their stochastic nature and cellular heterogeneity in cell cultures and organs. Depending on the biological question, a single-cell analysis can be essential.^1,2^ Unfortunately, a single cell's tiny amount of material complicates the measurements. Nevertheless, a few-cell analysis of preselected cell ensembles between two to ten cells can bring the desired parameters above the detection limit. Therefore, a ‘few-cell’ examination might be an alternative to a ‘real’ single-cell analysis. Particularly for multi-omics experiments, a few-cell approach might be more feasible.

Different strategies were developed for the minute sample amounts of single or few-cell analysis. Amplification technologies simplify the investigation of single-cell transcriptomes and genomes.^3,4^ Unfortunately, amplification is not possible for the proteome and metabolome. However, many metabolites are present in high copy numbers in the cell, and the sensitivity of the state-of-the-art instruments is sufficient for the detection by mass spectrometry (MS). A complication is the large chemical variability of metabolites. As a result, metabolomic studies are dominated by targeted approaches for specific biochemicals. However, untargeted metabolite screens would be possible if liquid-chromatography mass spectrometry (LC-MS) could analyze single-cell extracts.

Only highly expressed proteins can be measured on the few- or single-cell proteomics level by untargeted MS approaches.^5^ Alternatively, the single-molecule characterization power of electron microscopes provides a potential strategy for the unlabeled analysis of a single cell's proteome. Until recently, ‘visual proteomics’ was limited to detecting large protein complexes.^6–9^ However, we witness now a fast technological development, with the advent of lamella milling into vitrified cells^10–13^ or microfluidic lysis of individual cells and subsequent preparation of the cell proteome for electron microscopy.^14–17^ Besides that, fluorescent light microscopy and reverse-phase protein arrays (RPPA) using cognitive molecules such as antibodies are efficient, targeted approaches for single-cell analysis.

The acquisition of few-cell or single-cell data and information analysis is challenging. Many samples of few or single cells must be individually characterized, which requires high-throughput approaches and extensive bioinformatics for data interpretation. Correlative methods allowing the simultaneous acquisition of information of multiple domains, *e.g.*, the characterization of proteins and metabolites, can significantly improve data interpretation.

**Fig. 1:**
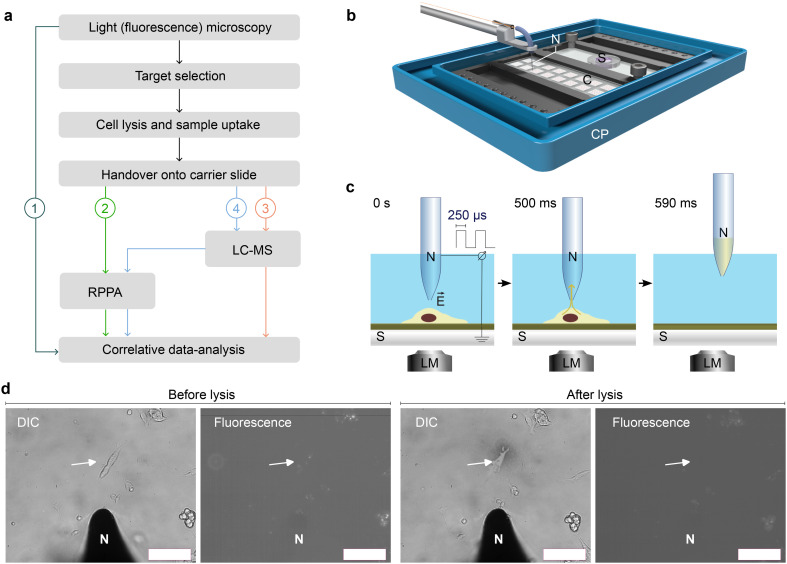
Workflow overview, single-cell lysis principles, and light microscopy using the cryoWriter setup. a) Overall workflow for the correlative analysis. Live cell imaging by (fluorescence) light microscopy (LM) is used for the structural and functional characterization of individual cells (1). The microscope is used for target selection, the monitoring of the single-cell lysis, and the cell contents' uptake. The cell lysate is dispensed onto a carrier slide for subsequent analysis by reverse-phase analysis (RPPA) and liquid-chromatography mass spectrometry (LC-MS, see Fig. 2 for details). The slides can either be analyzed by RPPA (2) or LC-MS (3) alone or in a combined mode (4), where first LC-MS is performed with subsequent analysis by RPPA. b) Light- and fluorescence microscopy imaging stage for live-cells, integration of the single-cell lysis setup, and handover system. In a live-cell incubator, cells are grown in a PDMS well on an ITO-coated slide (S), lysed and aspirated with a microcapillary electrode (nozzle, N), and spotted on the adjacent microarray slide (C). c) Adherent eukaryotic cells are grown on functionalized and electrically conducting ITO-coated glass slides (S). The cells are imaged using a (fluorescence) LM. An individual cell is located in the LM and lysed by electropulses (*E⃑*) between the ITO-coated slide and the electrically conductive microcapillary for single-cell lysis.^14^ Simultaneously, the lysate is aspirated in a volume of ≈3 nL into the microcapillary nozzle (N). Figure not in scale. d) Cell imaging and lysis monitoring by differential interference contrast (DIC) and fluorescence light microscopy. For a movie see also the ESI† Fig. S2. As an application example, undifferentiated cells (SH-SY5Y) were incubated with fibrillated α-synuclein, which was fragmented by freeze–thaw cycles and fluorescently labeled with an NHS-Alexa dye. This fluorescence signal guides and triggers the cell selection. The nozzle (N) targeted the cell for lysis by the combined forces of electroporation and friction by suction (see panel c). The arrow marks the target cell before and after lysis. Scale bars: 100 μm.

**Fig. 2:**
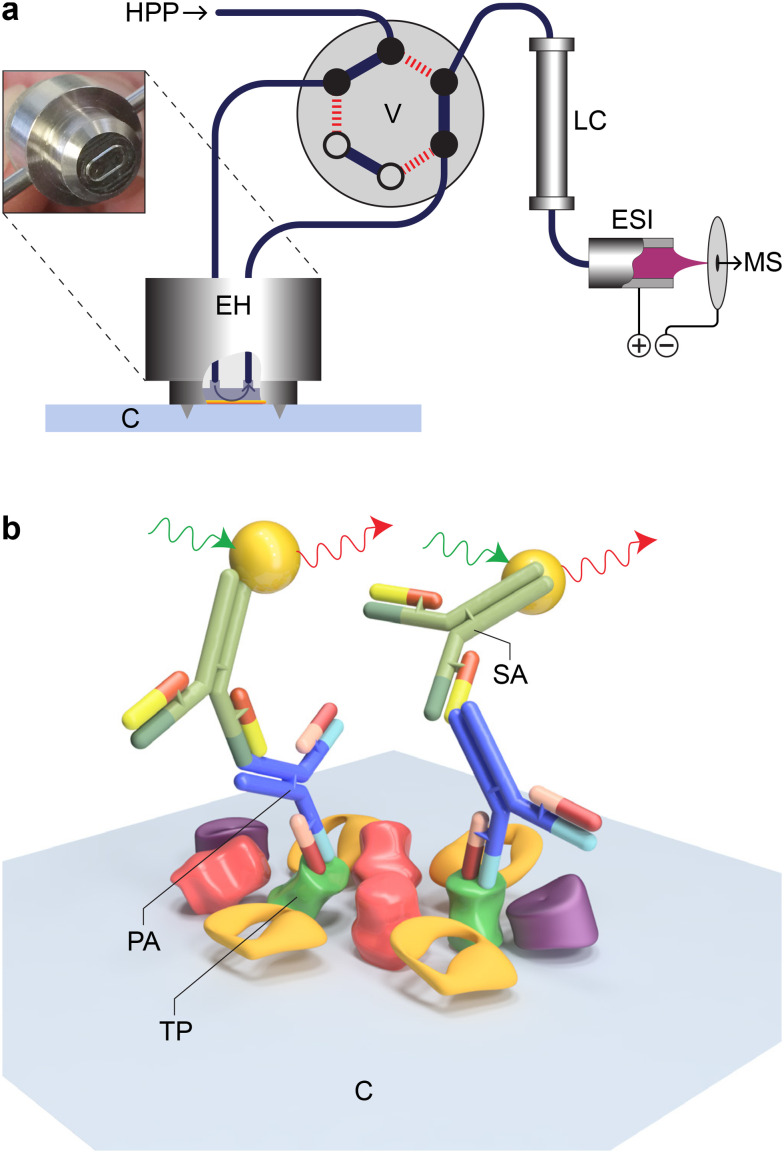
Principles of employed analysis technologies for the correlative multiomics analysis. a) High-pressure metabolite extraction for liquid-chromatography mass spectrometry (LC-MS). A high-pressure pump (HPP) presses the solvent *via* a six-port two-position valve (V) into the extraction head (EH), which is firmly pressed onto the carrier slide for tight sealing. The extraction head forms a small chamber at the deposition spot of the cell lysate. The stream is fed back to the valve into a liquid chromatography column (LC) and, after analyte separation into the electrospray for ionization (ESI) and mass analysis by the spectrometer (MS). As a result, a chromatogram is obtained, which can be for total counts (all analytes), or the analyte with a specific *m*/*z* ratio is analyzed. b) RPPA principles. The lysate spots containing the target protein (TP) are incubated with primary, target-specific antibodies (PA). Fluorescently labeled secondary antibodies (SA) bind to the primary antibodies' Fc domain.

Here, we present a single and few-cell analysis strategy for correlating visual features of cells (fluorescent light microscopy) to proteins (RPPA) and metabolites (LC-MS, Fig. 1a). We combined a light microscope with a single-cell lysis device, allowing first the visual selection of an adherent eukaryotic cell for subsequent lysis and uptake of the cell's content (Fig. 1b–d). A handover system enables the analysis of the cell lysate for metabolites by LC-MS (Fig. 2a) and target proteins by RPPA technology (Fig. 2b). The LC-MS and the RPPA analysis can be individually performed on the single-cell level. Furthermore, our data provide proof-of-concept measurements for few-cell experiments (2 cells to 10 cells) and demonstrate the feasibility of correlative single-cell analysis.

## 2 Experimental section

### 2.1 Cell preparation for single and few cell experiments

We tested the single-cell lysis with two different cell lines using marginally different, cell-specific proliferation media. The human, neuroblastoma-derived SH-SY5Y cells (SH-SY5Y Cell Line human, 94030304, Sigma, Switzerland) were grown in 1 : 1 MEME (minimum essential medium Eagle with earl salts and sodium bicarbonate (M2279-500ml, Sigma)) and HamF12 media (Nutrient Mixture F-12Ham, N8641, Sigma, Switzerland), GlutaMAX (35050-061, Gibco, Life Technologies, Switzerland), NEAA (non-essential amino acids, M7145, Sigma, Switzerland), and 10% FBS (16000-044, Gibco, Life Technologies, Switzerland). The human embryonic kidney cells (HEK293, 85120602, Sigma, Switzerland) were grown in DMEM + GlutaMAX (Dulbecco's modified Eagle medium, 61965-026, Gibco, Life Technologies, Switzerland) and 10% FBS. After three days of proliferation in 5% CO_2_ at 37 °C the cells were washed with phosphate-buffered saline (PBS), detached by adding trypsin-EDTA (0.05% trypsin, 0.53 mM EDTA; 25300-054, Invitrogen, Switzerland), diluted with proliferation medium and centrifuged for 5 min at 100 rcf. The supernatant was removed, and the pellet was resuspended in the medium. A small amount was further diluted and sent into proliferation again.

For single-cell lysis experiments, the cells were grown in miniaturized Petri dishes consisting of indium tin oxide (ITO) coated glass slides with bonded flat rings from poly(dimethylsiloxane) (PDMS, SYLGARD 184, Dow Corning, USA) forming small wells with a volume of 50 μL. The ITO-slides and the PDMS rings were stored in 70% ethanol. The rings were sonicated for 20 min at 35 kHz in a water bath to remove ethanol and promote further sterilization (Bandelin Electronics, Sonorex RK31, Germany). The ITO-slides were dipped in 100% ethanol and flame-sterilized. Immediately afterward, the PDMS rings were directly bonded to the still-warm slides. Subsequently, the micro-wells were dried in the flow hood under UV sterilization for 30 min. The wells were then treated with poly-l-lysine (PLL, P8920, Sigma, Switzerland) for 15 min. Finally, 10^4^ cells were loaded per well and grown in a total volume of 50 μL of cell-specific proliferation media in 5% CO_2_ at 37 °C for 2 d.

### 2.2 Transfer slide substrates

The polymer slide substrates subjected to testing included cyclic olefin copolymer (COC) (Microfluidic ChipShop GmbH, Germany), polyether ether ketone (PEEK) (Mechanical workshop at Hoffmann-La Roche AG, Switzerland), and polytetrafluoroethylene (PTFE) (Mechanical workshop at Hoffmann-La Roche AG, Switzerland). LC-MS experiments comparing these slides are presented in Fig. S6.† Furthermore, correlative measurements were performed using NHS-functionalized glass slides (NEXTERION® Slide H, Schott, Germany). Initial RPPA experiments were performed using nitrocellulose-coated (NC) slides (16-pad FAST slide Maine Manufacturing, Sanford, Maine, U.S.A.) to test the hand-over system (Fig. S1†).

### 2.3 Single cell picking

Cell picking and lysis were performed with the cryoWriter^14,16,18,19^ setup (Fig. 1). The cell slides and NHS-functionalized transfer slides were placed in a cell incubator (INUBG2ETFP-WSKM, Tokai Hit, Shizuoka, Japan) in 5% CO_2_ at 37 °C mounted on top of an inverted microscope (Axiovert 200, Zeiss, Germany) with a motorized stage (H117EIL5, Prior Scientific, UK), see also Fig. 1b. Two metal screws electrically grounded the ITO-coated cell slides as described before.^14,15^ A micro-capillary (Picotip, New Objective Inc., USA) with a conductive coating (20 nm 10% Ti–90% W, 200 nm Pt) was implemented for cell picking, electrical cell lysis, and lysate deposition. The micro-capillary was mounted on three linear stages (*x*, M-414.3PD; *y*, *z*, M-404.2PD, Physik Instrumente, Germany) and was connected to a syringe (1701 LT SYR, Hamilton, USA) on a high-precision syringe pump (neMESYS, Cetoni GmbH, Germany). The system was primed with demineralized, degassed water (water for Molecular Biology, Merck Millipore, Germany) with almost zero dead volume for precise pipetting. A function generator (33220A, Agilent, Switzerland), combined with a voltage amplifier (F20A, FLC Electronic AB, Sweden), was connected to the conductive coating of the micro-capillary. Immediately before the lysis experiments, the cell medium was removed, and the wells were washed three times with PBS. Cells were selected with a light microscope. The capillary was positioned 10 μm above the slide over the target cell. Three square pulses (*f* = 2 kHz, *A* = 19 V) were generated for the electroporation of the cell. After 500 ms, 3 nL containing the cell lysate were aspirated. To ensure the lysis of a single cell, the cell confluence was maintained between 10% to 30%. As the electric field decreases with an inverse squared distance behavior, this range allows for sufficient spacing between cells, preventing neighboring cells from being lysed or taken up when targeting a specific cell. Subsequently, the lysate was applied to the transfer slides in a distinct pattern and dried under an argon atmosphere. After spotting, the slides were transferred to the LC-MS while still under an argon atmosphere, enclosed within a lightproof tube. The whole instrument was controlled and automated by an in-house developed software based on the openBEB framework.^20^

### 2.4 Seeding experiments

Synthetic α-synuclein fibrils were grown in 660 μL of Dulbecco's phosphate buffered saline with MgCl_2_ and CaCl_2_ (DPBS, D8662-500ML, Sigma, Switzerland) and 0.05% sodium azide (S2002, Sigma, Switzerland) at a starting monomer concentration of 0.498 mg mL^−1^ over seven days on an Eppendorf shaker (Thermomixer compact, Vaudaux-Eppendorf AG, Basel, Switzerland) at 37 °C and 700 RPM. Synthetic fibrils were harvested once the solution became opaque and truncated to viable seed length (50 nm) with a 12-snap-freeze–thaw cycle.

α-Synuclein seeds were covalently modified with Alexa Flour 488/598 carboxylic acid succinimidyl ester (A20000, A20004, Invitrogen, Switzerland). Two batches of 330 μL seeds were placed in 33 μL 1 M sodium bicarbonate (Sigma, S5761-1KG, Switzerland) before being dissolved in 100 μL dimethyl sulfoxide (DMSO, Sigma-Aldrich, 276855-100ML, Switzerland). Additionally, 30 μL of the ester dye was mixed with the seeds before being added to a Slide-A-lyzer 3.5K dialysis cassette (Thermo Scientific, 3500 MWCO, 11859410, Switzerland). This cassette was incubated at room temperature for 2 h in 400 mL PBS (P4417-100TAB, Sigma, Switzerland). The buffer was switched out for another 400 mL of PBS buffer and left covered overnight for 18 h. These labeled α-synuclein seeds were added (2.5 μL per 1 mL media/cell solution) to SH-SY5Y cells after the cell pellet was re-suspended in proliferation media (as described above).

### 2.5 Batch lysate preparation (SH-SY5Y)

The cells (SH-SY5Y Cell Line human, 94030304, Sigma, Switzerland) were prepared as described above (subsection 2.1). After centrifugation, the cell pellet was washed and dissolved in 25 mM HEPES–KOH, 5 mM MgCl_2_ at pH 7.5, and the cell concentration was determined by counting (6 × 10^6^ cells per mL). Finally, the cells were lysed by sonication (UP200St, Hielscher, Germany) for 40 s (*P* = 200 W, *f* = 26 kHz).

### 2.6 Batch lysate preparation (LUHMES)

Lund human mesencephalic (LUHMES) cells were grown and differentiated according to a published protocol.^21^ For the preparation of batch lysate, the cells were washed with PBS and lysed using a lysis buffer (50 mM Tris buffer (Sigma, Switzerland), pH 8.2, 75 mM NaCl (Sigma, Switzerland), 8 M urea (Sigma, Switzerland), protease inhibitor cocktail (complete mini, Roche, Switzerland), 1 mM phenylmethylsulfonyl fluoride (PMSF) (Sigma, Switzerland), 10 mM sodium pyrophosphate (Sigma, Switzerland), 1 mM sodium orthovanadate (Sigma, Switzerland), 1 mM β-glycerophosphate (Sigma, Switzerland)). The batch lysate was aliquoted and stored at −20 °C. Before use, the aliquots were diluted with water to a final concentration of ≈1250 cells per μL.^22^

### 2.7 Liquid-chromatography mass-spectrometry

The system consists of a thin-layer chromatography interface (TLC-MS Interface 2, CAMAG, Switzerland), which has been connected to a binary high-performance liquid chromatography (HPLC) pump (1100, Agilent, USA) and a six-port, two-position valve. A water-resistant C18 column (Cortecs T3 2.7 μm, 2.1 × 100, Waters, USA) was used. The TLC-MS interface's extraction head was modified with a 3D-printed polypropylene cap (Prusa i3 MK3, Prusa Research, Czechia & PP 55950, Verbatim, ROC) to ensure effective sealing with the glass transfer slides. The configuration is illustrated in Fig. 2a. The interface was established between a high-resolution mass spectrometer (LTQ-Orbitrap XL, Thermo Scientific, USA) as well as a low-resolving triple quadrupole system (6460, Agilent, USA) equipped both with an electrospray ionization (ESI) source (Thermo Scientific, USA). For non-targeted analysis and initial evaluation a gradient starting at 100% water containing 0.1% formic acid holding at this conditions for 60 s followed by a linear gradient for 6 min to 100% methanol containing 0.1% formic acid at a flow rate of 0.5 mL min^−1^ was used. For targeted analysis, the gradient was isocratic, staying at 100% water containing 0.1% formic acid for 1 minute followed by a linear gradient of 2 min to 100% acetonitrile containing 0.1% formic acid. The multi-reaction monitoring (MRM) modes used were 148 → 84.6/collision energy (CE) = 14 for glutamic acid; 147 → 130/CE = 15 for glutamine; 154 → 137/CE = 2 for dopamine. Nicotine was used as a standard to validate alignment and system performance. Sensitivity in the low pg (Orbitrap) to fg (triple quadrupole) range was achieved for all standards. All reference standards were purchased from Sigma Aldrich (Switzerland). Test samples for system characterization (all Sigma Aldrich, Switzerland) are listed in Table 1. Solvents used for experiments were ddH_2_O, methanol, isopropanol, and acetonitrile, all supplemented with 0.1% formic acid (HPLC grade, Sigma Aldrich, Switzerland).

**Table 1:** List of the tested metabolites and drugs. A sample mix was spotted on COC slides and eluted with the extraction interface into the LC-MS. For comparison, the same sample mix was directly injected into the LC-MS instrument. The samples were measured in full scan mode. Additional sensitivity could be obtained by measuring in a targeted MRM mode. Retention times as well as the limit of detection (LOD) for directly injected samples and samples eluted from the slide are shown. A signal-to-noise ratio of three was used to estimate LOD. The log *P* value is a measure of a compound's lipophilicity or hydrophobicity. The tested molecules are spread across a broad range of log *P* values

Compound	[M + H]^+^	log *P*	Concentration [μg mL^−1^]	Retention time [min]		LOD [pg]	
				Injected	Slide	Injected	Slide
Alanine	90.0544	−2.85	0.488	0.44	0.67	24.40	24.40
Aspartate	134.0442	−3.89	0.192	0.45	0.67	34.80	9.70
Glutamic acid	148.0599	−3.69	0.497	0.46	0.68	24.84	24.90
Valine	118.0857	−2.26	0.250	0.67	0.85	12.50	12.50
Glutamine	147.0759	−3.64	0.492	0.68	0.67	24.25	24.60
Nicotine	163.1224	1.10	0.101	0.83	0.83	5.05	5.05
Adenosine	268.1035	−1.20	0.789	2.26	—	39.40	—
Phenylalanine	166.0857	−1.38	0.410	3.21	—	24.60	—
Acetaminophen	152.0701	0.46	0.392	4.45	4.55	39.20	19.60
Sulfadimethoxine	311.0803	1.08	0.725	6.92	7.02	36.25	36.30
Carbamazepine	237.1017	2.30	0.153	7.47	7.55	30.60	7.65
Testosteron	289.2157	3.32	0.112	8.56	8.63	56.00	22.40
Diclofenac	296.0234	3.90	0.228	9.48	9.57	22.80	91.20

### 2.8 Immunolabeling for reverse phase protein array (RPPA)

First, the slides were treated with a blocking solution (Roti-Block, Carl Roth AG, Switzerland) for 90 min. Subsequently, the slides were incubated with primary antibodies (mAb mouse anti-actin IgG CLT 9001, pAbs rabbit anti-GAPDH IgG ab37168) each at a concentration of 1 μg mL^−1^ in washing buffer (10 : 1 mixture of PBST 0.05% and blocking solution) for 20 h. Afterward, the slides were washed for 30 min in washing buffer and subsequently incubated with secondary antibodies for 60 min. For the infrared scanner (Odyssey 9120, Li-Cor, USA), donkey anti-rabbit IgG 925-68073 and donkey anti-mouse IgG 926-32212 at a dilution of 1 : 10 000 in washing buffer were used. For the fluorescent microscope (Axiophot, Zeiss, Germany), Alexa 488 donkey anti-rabbit IgG 711-545-152 and Cy3 donkey anti-mouse IgG 715-165-151 at a dilution of 1 : 1000 or 1 : 3000 respectively in washing buffer were used. The slides for the fluorescent microscope were subsequently covered with Mowiol 4-88 (Carl Roth AG, Switzerland).

### 2.9 Data analysis

Final data analysis and generation of graphs was performed using Python 12 in Jupiter notebooks using the libraries numpy (1.24.4), matplotlib (3.8.1), pandas (1.5.3), SciPy (1.11.1), and SciencePlots (2.1.0). For image processing of the fluorescence signals of the RPPA raw data, Fiji (ImageJ)^23^ was used. Initial data analysis for the LC-MS data was performed using Xcalibur (Thermo Fisher, USA) and Mass Hunter (Agilent, USA). The Thermo-Fisher raw files were converted using msconvert (ProteoWizard)^24^ to the mzML format and further processed in Python using the pyopenms (3.0.0)^25^ library.

## 3 Results

We combined a light microscope with a single-cell picker device and a hand-over system,^14,16,18,22^ which allows cell-lysate deposition onto carrier slides as an array for subsequent multiplexed quantification (Fig. 1b and c). The workflow enables correlative analysis by (i) (fluorescent) light microscopy (LM, Fig. 1d) before and during cell lysis, (ii) LC-MS, and (iii) RPPA (Fig. 1 and 2).

Whereas LM monitoring is used for the structural characterization, selection, and lysis of target cells, LC-MS and RPPA use the sample arrays on the carrier slide for subsequent analysis. For the metabolite analysis by LC-MS, the low-molecular-weight analytes are extracted from a selected cell-lysate spot by a solvent using a modified TLC-MS interface (Fig. 2a and ESI† H) directly connected to an LC-MS. This configuration enables targeted or untargeted analysis. An RPPA immunoassay was used to detect protein for targeted proteomics. Thereby, the whole slide was incubated with a primary and secondary antibody, and the fluorescence signal of the secondary antibody was counted (Fig. 2b).

### 3.1 Light microscopy, single-cell picker, and hand-over system

The combined forces of electroporation and suction (Fig. 1c) were used to lyse and pick adherent eukaryotic cells under the supervision of a light microscope.^14^ The cell growth chamber (Fig. 1b) allows the cultivation of cells in a humidified, temperature-controlled, and CO_2_-enriched environment. This system allows imaging of the cells by fluorescence and DIC light microscopy.

For electroporation, both the microcapillary and the cell growth substrate must be electrically conducting. We used grounded ITO-coated glass slides suitable for (fluorescence) LM, which were functionalized with poly-l-lysine for cell growth. The pulled microcapillary with an inner diameter of 100 μm and an apex diameter of 30 μm were coated with an 20 nm 10% Ti–90% W and an 200 nm Pt layer. The target cell is destabilized by electroporation (3 × 0.5 ms, amplitude 19 V, nozzle-slide distance ∼10 μm). Simultaneously, a volume of 3 nL is aspirated, sucking the cell lysate into the microcapillary. The whole cell-lysis process takes less than one second; see Kemmerling *et al.*, 2013 (ref. 14) for a detailed discussion, and the ESI† Movie S2.

We used cell cultures with a confluency of 10% to 30%, allowing the selection of individual cells. The lysis procedure did not influence neighboring cells, which could divide and proliferate afterward (see Fig. S3†). Occasionally we observed some bubbling after the lysis process at the nozzle tip due to electrolysis of water molecules, which is catalyzed by the Pt coating.

### 3.2 Assay development and benchmarking with batch cell lysate

For the LC-MS and RPPA analysis, carrier slides were utilized for the transfer and subsequent processing. These slides' mechanical and physicochemical characteristics are pivotal in facilitating the correlative analysis by LC-MS and RPPA. Despite the sealing properties with the extraction head, we tested the release of chemical compounds from the substrate material by LC-MS as shown in Fig. S6.† We found that COC has good sealing properties, minimally releases chemicals, and retains proteins well for the subsequent analysis by RPPA; therefore, we used COC slides for the initial assay development.

**Fig. 3:**
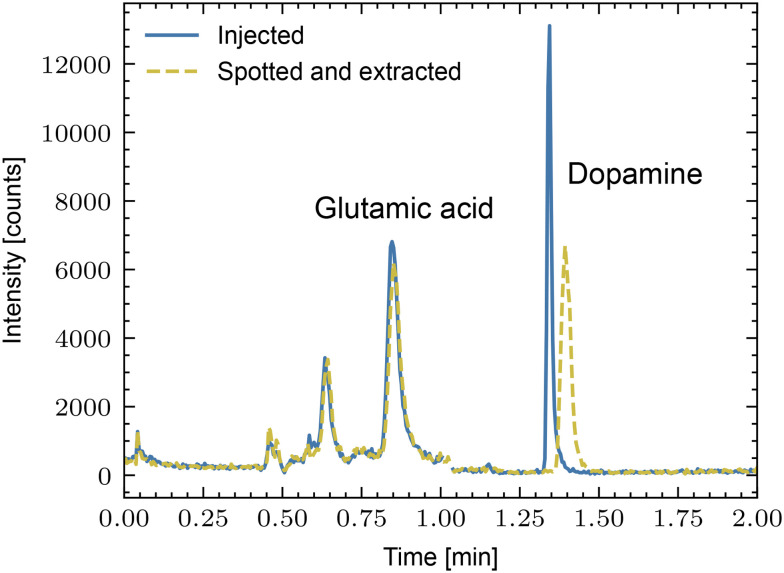
Comparison of directly injected (solid line) with spotted and extracted (dashed line) metabolites. As test samples, the neurotransmitters glutamic acid and dopamine were used. Note that the retention times depend on the LC columns used.

Another question is the stability of metabolites after dispensing onto the carrier slides, mainly when the slides must be stored after the single-cell lysis and transported to the mass spectrometer. Therefore, we compared small analytes directly injected into the LC-MS with spotted and extracted samples. First, we compared glutamic acid and dopamine by performing LC-MS in multiple reaction monitoring (MRM) mode Fig. 3. A mixture containing 52 pg glutamic acid and 72 pg dopamine was directly injected into the LC-MS instrument, and the identical amount of sample was spotted on COC and extracted with the extraction interface. As expected, the retention time (RT) was shifted, particularly for dopamine. Additionally, peak broadening was observed for the dopamine peak of the COC-eluted sample. However, the peak area remained about the same.

Additionally, we performed experiments with a mixture of typical metabolites covering the entire range of log *P* values (a measure of how hydrophilic or hydrophobic a molecule is, Table 1). We spotted, extracted, and analyzed the mixture by LC-MS. For comparison, the same amount of the mixture was directly injected into the LC-MS system. Note that these experiments were performed without a protecting atmosphere, and the analytes were exposed to the oxygen of the surrounding air. A comparison of the limit of detection (LOD) shows that around 50% of the tested analytes had a comparable (or even better) LOD. However, some metabolites were significantly degraded on the slide, particularly compounds with aromatic rings as shown in Fig. 3, which was apparent when comparing glutamic acid and dopamine. An Ar-atmosphere for transport and storage used for subsequent experiments efficiently protected the analytes.

We conducted experiments to test the effectiveness of our workflow for analyzing single or few cells using a dilution series of SH-SY5Y batch lysate. The cells were cultured in proliferation media for three days, washed with PBS, and detached using trypsin-EDTA. After diluting with proliferation medium, the cells were centrifuged, and the resulting pellet was dissolved. The cell concentration was measured by counting, and then the cells were lysed by sonication. Using the handover system, we applied lysate amounts equivalent to 1, 3, and 9 cells to carrier slides. Subsequently, we analyzed the sample spots using LC-MS followed by RPPA for protein detection.

**Fig. 4:**
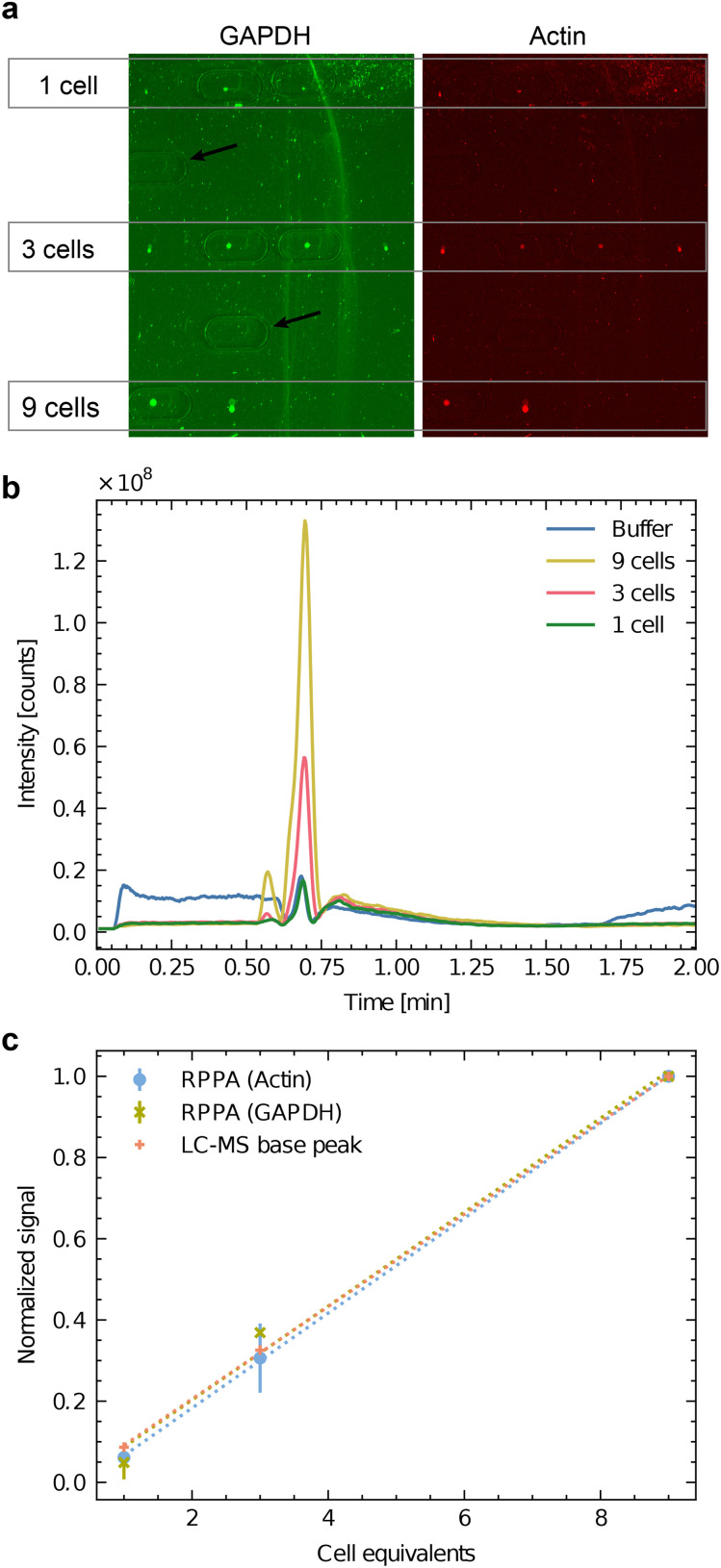
Correlative analysis of batch cell lysate by RPPA dot-blots and HPLC-MS. SH-SY5Y batch cell lysate was dispensed to an equivalent of 1 cell (3 nL), 3 cells (9 nL) and 9 cells (27 nL). a) RPPA analysis of GAPDH (green) and actin (red) as two abundant test proteins. Four experiments were dispensed for the equivalent of one and three cells (*n* = 4), and two lysis experiments were dispensed for the equivalent of nine cells (*n* = 2). Note the imprints of the HPLC-MS interface extraction head are visible as oval lines. The black arrows indicate blank positions where the buffer was dispensed as a negative control. (b) Base peak chromatogram of the untargeted LC-MS experiment on the Orbitrap instrument. Mass range 
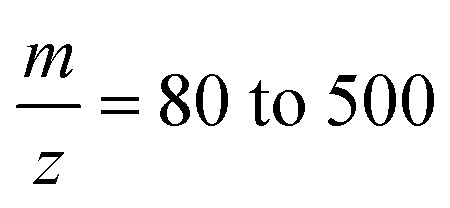
. The spectra of ESI† I depict apparent differences between buffer and cells in a semi-quantitative matter. c) Correlation between the two normalized RPPA signals (actin and GAPDH) and the normalized LC-MS signal. For the normalization, the averaged signals for 9 cells were used. The RPPA signals of panel a) were quantified, and the global background was subtracted. For the LC-MS analysis, the base-peak area was measured (panel b).

Fig. 4a shows the COC carrier slide after metabolite extraction and RPPA analysis. The protein's signals of the dispensed sample spots are visible and represent the sample spots utilized for the multi-omics analysis. The imprints made by the extraction head are apparent (black arrows indicate the extraction locations of the blanks at locations without cell lysate deposition). Immunofluorescent labeling by RPPA for two abundant proteins (GAPDH in green and actin in red) are visible in Fig. 4a. The results show the capability to detect actin and GAPDH down to the equivalent of a single cell, even after metabolite extraction.

Fig. 4b shows the LC-MS chromatogram (total counts of the MS signal between 
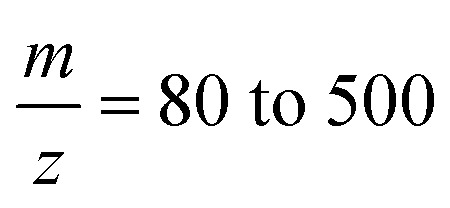
) of the extracted metabolites of the spots shown in panel Fig. 4a. The untargeted mode showed that this interface could transmit several metabolites for LC-MS analysis even at a low number of cells. Quantifying the dominating peak detected after 40 s revealed a roughly linear relationship between the peak area and the cell equivalents of the applied sample spots (Fig. 4c).

Furthermore, Fig. 4c compares the area of the dominating LC-MS peak in the chromatogram with the normalized RPPA signal intensity for GAPDH and actin. Interestingly, the ratio of the signals roughly stays constant between the three different read-out channels, indicating that the workflow is suitable for a correlative multi-omics analysis. As expected, neither the RPPA nor the LC-MS are quantitative, but the method allows measuring the relative abundance of the target analyte. It enables direct comparisons of small cell populations.

**Fig. 5:**
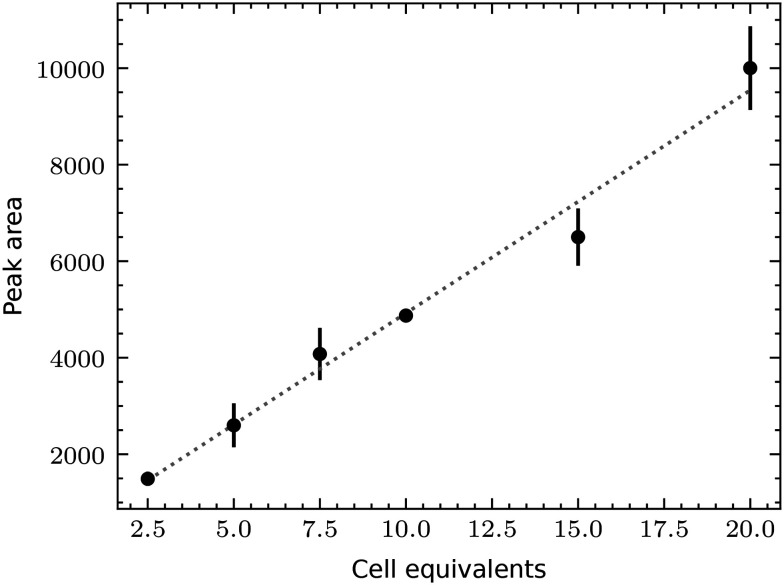
Targeted quantification of glutamic acid in LUHMES batch cell lysate by multi-reaction monitoring (MRM) on the Orbitrap configuration (linear trap). The smallest measured spot corresponds to approximately 2.5 cells using the handover system shown in Fig. 1b and 2a.

The proposed workflow also allows the targeted semi-quantitative measurement of the relative abundance of metabolites. Fig. 5 presents the detection of glutamic acid in dependence of the dispensed cell equivalents. The integrated peak area for glutamic acid shows an explicit linear dependency of the dispensed cell lysate amount. The most minor successfully measured spot corresponded to approximately 2.5 cells.

### 3.3 Single cell analysis for protein detection by RPPA

**Fig. 6:**
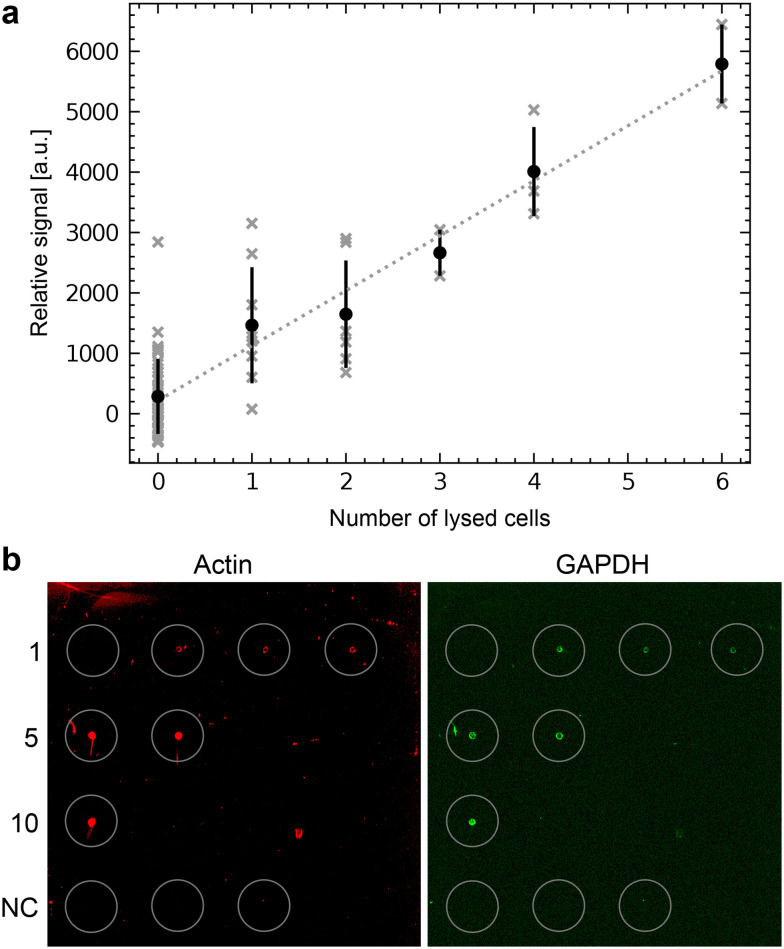
Single-cell RPPA. a) One to six HEK cells were lysed, aspirated and dispensed as spots on an NC-pad on the carrier slide. The slide was scanned after incubation with an anti-actin antibody and a fluorescently labeled secondary antibody. The total fluorescence intensity was plotted against the number of lysed cells dispensed in each spot. The average intensities and the standard deviation are indicated in black to show the variation. The individual cells are shown in gray. A linear regression is shown as a dashed line. b) Single-cell RPPA dot-blot using an NHS-functionalized glass slide for covalent immobilization of the proteins. The left panel shows the detection of actin (in red), and the right panel of GAPDH (in green). Single cells were individually lysed and dispensed. This cycle was repeated for every spot for 1, 5, and 10 cells. Cumulated volumes of 3 nL, 15 nL or 30 nL of lysate were dispensed, depending on the number of lysed cells. The number of individually lysed cells per spot is indicated in the left column. The last row depicts the negative control (NC), where 10 nL, 20 nL or 30 nL of the buffer surrounding the cells were dispensed.

We first tested the single-cell analysis of adherent eukaryotic cells separately to detect proteins by RPPA without metabolite extraction for LC-MS. We used HEK cell cultures grown on functionalized ITO slides. We applied the single-cell lysis setup for the cell targeting using the light microscope and the subsequent lysis and aspiration of the target cell. Subsequently, the cell lysate was dispensed onto NC-coated carrier slides. We dispensed the lysate of between 0 and 6 cells and quantified the fluorescence signal of the immunolabeled actin proteins (Fig. 6a). The average fluorescence signal shows an explicit dependency on the number of lysed cells, which is roughly linear. As expected from single-cell analysis, we also noticed a considerable variation in the individual cells' signal as indicated by the x-markers. Additional experiments compare individually lysed cells using the single cell lysis module with batch cell lysate of HEK cells confirm the roughly linear relationship between the detected signal and number of dispensed cells or cell equivalents, respectively (Fig. S5†). Note the high dynamic range of the RPPA signal, although the signal reaches saturation above >10^4^ dispensed cell equivalents.

### 3.4 Single cell analysis for metabolites and correlative single-cell characterization

**Fig. 7:**
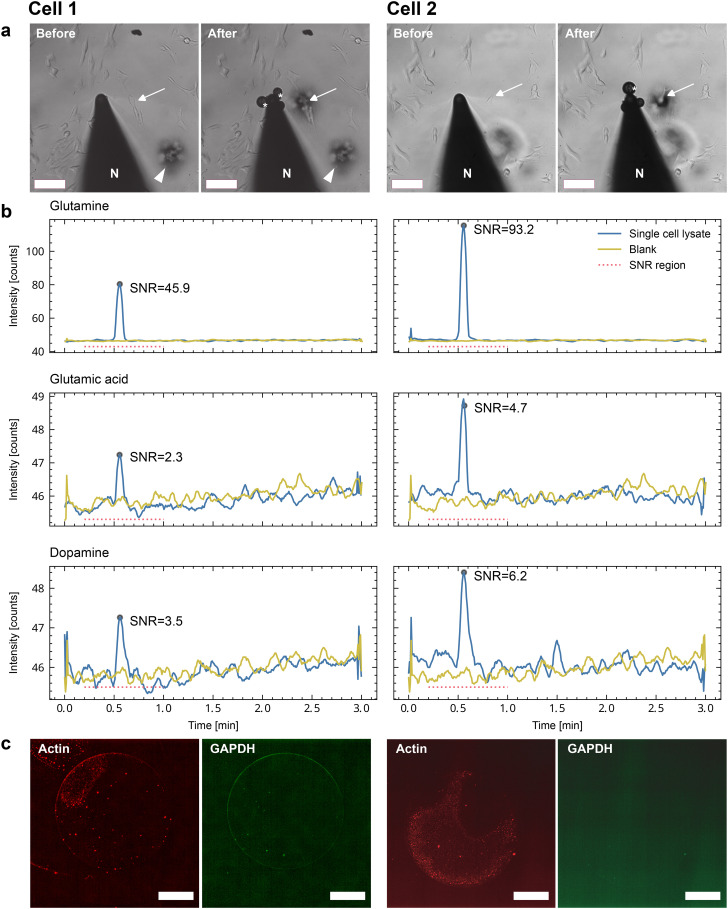
Correlative single-cell imaging and combined LC-MS and RPPA analysis. Two individual cells were lysed (cell 1 & 2) and the cell lysate was dispensed on carrier slides for subsequent analysis by LC-MS followed by RPPA (Fig. 2). Note the limitations of the correlative RPPA analysis after LC-MS on the single-cell level. a) Light-microscopy images before and after lysis of a target cell as indicated by the white arrows. For single-cell lysis, the microcapillary nozzle (N) is moved above the target cell and the the adherent cell is lysed by the combined forces of electroporation and shear stress during the 3 nL aspiration. The lysis is monitored in the light microscope by moving the nozzle back. After the successful lysis, the cell contents are dispensed on the carrier as shown in Fig. 1. Scale bars: 100 μm. b) Single-cell HPLC-MS analysis of glutamine, glutamic acid, and dopamine (important metabolites in neurons). The extraction was performed independently at two spots with single-cell lysate and a spot without cell lysate (blank, negative control). The base peak eluate was analyzed for the corresponding 
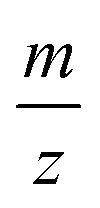
 ratios. The signal of the metabolites is represented in blue, and the blank is shown in yellow (same negative controls for both individual cells). The signal-to-noise ratio (SNR) is indicated for all six peaks, and the region for the peak search and baseline information needed to determine the SNR is shown as a dotted red line. c) Dot-blots of the two cells after metabolite extraction. The signals for actin (red) and GAPDH (green) are shown. In contrast to the dilution series of batch cell lysate, the signal is barely visible after metabolite extraction. Note the coffee-ring (drying) effect. Scale bar: 120 μm.

Fig. 7a–c depicts the correlative characterization of two individual cells. We lysed individual SH-SY5Y cells under the supervision of a light microscope and transferred the cell lysate onto a sample carrier slide. Panel a shows the light-microscopy images of the selected cells before and after cell lysis and uptake into the microcapillary. Subsequently, metabolites were extracted as shown in Fig. 2a and analyzed by LC-MS. The detection of three different metabolites is reported in panel b. After metabolite extraction, immunostaining and RPPA analysis were performed to detect two housekeeping proteins (panel c).

The workflow (Fig. 1 and 2) enables the qualitative detection of metabolites after extraction by LC-MS. Fig. S7a† shows the detection of glutamine and glutamic acids extracted from single-cell lysate deposits on COC polymer slides. Unfortunately, the COC and the other previously tested unfunctionalized polymers did not efficiently retain the proteins from an individual cell during the LC-MS metabolite extraction. As a result, the sensitivity for the subsequent detection of the proteins by RPPA on the single-cell level was significantly degraded (ESI† S7b). Therefore, we tested NHS-functionalized glass slides, allowing the covalent immobilization of proteins *via* their primary amines such as lysines. Fig. 6b shows an RPPA dot-blot from individually picked cells on a functionalized glass slide before metabolite extraction. The signal-to-noise ratio of single-cell RPPA dot-blot with functionalized carriers was better than with COC slides and comparable to NC-coated pads, which were, in our hands, unsuitable for the metabolite extraction process. A particular advantage of functionalized pads was the better concentration of proteins at the dispensing spot.

LC-MS analysis was conducted on a triple quadrupole LC-MS system employing the modified TLC interface, as illustrated in Fig. S8.† This head modification enables proper sealing of the extraction head on the NHS-functionalized carrier glass slides. The extraction of metabolites from the carrier slide was achieved using water containing 0.1% formic acid. Fig. 7b shows the MRM chromatograms of glutamine, dopamine, and glutamic acid. Additionally, a baseline is shown with eluate from a carrier slide region without cellular deposit (negative control, blank). We estimated the signal-to-noise ratio by comparing the LC-MS peak height with the standard deviation of the blank (the dotted red line indicates the region). Whereas the SNR of the glutamine and dopamine signals allow a semi-quantitative comparison, the SNRs of glutamic acid are significantly lower and close to the limit of being interpretable, taking an SNR threshold of >3.

Fig. 7c shows the corresponding dot in the RPPA analysis for actin (red) and GAPDH (green) after LC-MS metabolite extraction. Integration shows that the proteins are detected and exhibit an SNR of 1.1. This SNR is too low for a qualitative interpretation. There are three reasons for this low SNR: first, the fluorescent camera operated close to its detection limit. Second, we observe a large deposition area of the droplet. Notably, a “coffee-ring” effect is visible during the drying process after sample deposition. This effect leaves a large area without significant cell content, accumulating almost exclusively toward the droplet's edges. Thirdly, some proteins are still lost during extraction despite using functionalized slides.

## 4 Discussion

We present a method and setup for the correlative analysis of single and few cells by combining three different analysis channels. First, the cells are characterized by light microscopy, providing structural information and, if applicable, a fluorescence signal. Second, LC-MS analysis allows the untargeted and targeted study of metabolites on the single-cell level. Third, RPPA enables the targeted quantification of proteins. Whereas the current implementation of the setup allows single-cell analysis by combining light microscopy with LC-MS or RPPA, the combination of all three analysis methods currently works for a few-cell amounts of 3 cells to 10 cells. As discussed below, future amendments in the workflow can bring the correlative analysis for all three modalities to the single-cell level.

Notably, the workflow allows the preselection and targeting of adherent eukaryotic cells by DIC and fluorescent light microscopy. Thereby, the fluorescent signal can be used for the selection or as a trigger to initiate lysis of the target cell at a specific biological event (Fig. 1d). Importantly, such a preselection of cells enables the lysis and take up of multiple cells, enabling the ‘few cell analysis’ of cells in a similar state.

Why did we choose the presented electroporation method for single-cell lysis? First, it is an easy-to-implement method that directly and physically lyses and aspirates individual cells by combining electroporation and shear forces. No denaturing chemicals are employed, which could interfere with LC-MS analysis, and protein structures are conserved.^14^ Secondly, the lysis spot is relatively small (<100 μm), and the entire process is fast (<1 s). Third, we envisage the application of electroporation for manipulating single cells, *e.g.*, by diffusing effector molecules into electroporated cells. Depending on the investigated biological system, the handover system and analysis strategies (LC-MS, RPPA) can also be combined with other lysis methods.

Disadvantages are that the cell must be grown on a conducting substrate (in our case functionalized, ITO-coated microscopy glass slides), that the Pt-coating of the microcapillary can wear off with time, and that the lysis location temporarily heats (approximately by 20 K for few ms, see Kemmerling *et al.*, 2013 (ref. 14) for a discussion). We do not expect a brief and moderate temperature increase to impact the stability of metabolites or proteins, and our previous work showed no effect on native protein structures or enzymatic activities.^14,16,17,22^ Currently, we use a Pt-coating of the microcapillary. Pt catalyzes the electrolysis of water molecules; sometimes, the gas molecules accumulate at the nozzle tip as tiny bubbles. This effect could be mitigated using better-suited conductive material to coat the capillary, such as Ag/AgCl.

An important question is the affected area of the single-cell lysis. Since the electric field decreases with an inverse squared distance behavior, spacing of ≈50 μm between cells is sufficient for the electroporation of a single cell without affecting its neighbors. Such spacing is typically found at cell confluences of 10% to 30%. However, local variations in the dielectric constant (*e.g.*, in the media and cells) can influence the shape of the electric field. Our cell lysis workflow takes an image of the lysis location before and after cell lysis, which documents the lysis efficiency and potential effects on neighboring cells (see Fig. 1d). We tested the medium-turn effects of the lysis process on neighboring cells (Fig. S3†). We lysed a cell, gave the remaining cells a recovery time of 12 h, and imaged the exact location again in the light microscope. Neighboring cells can still divide and proliferate, documenting the cell's health.

To prevent cross-contamination of neighboring cells, the surrounding buffer can be diluted with a push of system liquid, such as PBS buffer or ultrapure water, immediately before cell lysis. This dilution visibly displaces potential contaminants, such as cell debris. Furthermore, using ultrapure water induces an osmotic shock, further destabilizing the cell if needed.

Notably, the method allows the preparation of many samples or individual cells in parallel by using array spotting of the cell lysate. Few-cell and single-cell research must reach appropriate statistics to detangle cellular biological noise, as seen in Fig. 6. The current prototype implementation of the cell lysis instrument using the openBEB framework^20^ can be automatized by a macro subsystem but still needs manual control and interventions. The extraction of the metabolites for LC-MS is entirely manual. With an additional *x*–*y* motorized stage, the LC-MS sampling of the dispensing spots could be easily automated.

The lack of automation has two main consequences: fewer samples (cells) can be processed quickly, and unstable biomolecules (*e.g.*, sensitive metabolites like dopamine) are lost during slow processing. The latter issue can be mitigated by providing a protective atmosphere and cooling the target slides during preparation. A new prototype will allow full automation in the future. Combined with an automated TLC interface, this new version would significantly increase the throughput of this method, making metabolite extraction the rate-limiting step and reducing the time to analyze an array spot to ≈120 s.

An increased speed would enable the collection of large datasets in a reasonable amount of time, helping to overcome the issue of biological noise and allowing for the exploration of cellular heterogeneity. The data collected with the current setup are insufficient for making statistically backed models; instead, they demonstrate the feasibility of correlative measurements and provide a proof of concept.

The workflow allows the single-cell analysis by LC-MS for the tested metabolites. The LC-MS system can be configured for targeted detection (Fig. 7) or in an untargeted mode (Fig. S9†). Even if the data does not allow for direct quantification in a targeted configuration, signals of the metabolites can be detected. Quantifying these metabolites would be feasible using newer LC-MS systems with significantly improved sensitivity available on the market.

How far are we from robust multiplexed measurement using all three modalities for single-cell analysis? For light microscopy, single-cell analysis is the standard; however, not for LC-MS and RPPA. We demonstrate that we have reached the single-cell level for the targeted metabolomics and proteomics for these analyses. However, the current configuration of the setup does not reach single-cell sensitivity in a correlative configuration for the RPPA after LC-MS. One of the reasons is the loss of proteins during the extraction of metabolites. A workaround we tested is the use of primary amine reaction slides. These slides improve the sensitivity for protein detection but might restrict the detection of metabolites with primary amines. Another challenge we have encountered is the ‘coffee-ring effect’, which leads to an inhomogeneous sample drying on the carrier slide. We propose the following solution: a Peltier-controlled stage allows for sample dispensing at the dew point temperature onto the slide, followed by a short temperature gradient to evaporate the sample liquid, effectively preventing the ‘coffee-ring effect’ (data not shown). This would also allow the primary amines of the proteins to react with the NHS groups of the functionalized glass slide before the sample spot is dried by increasing the temperature. Furthermore, we envisage a patterned carrier slide with predefined reactive spots surrounded by a hydrophobic surrounding, which prevents the spread of the dispensed single-cell sample.

## 5 Conclusions

Our workflow combines three different analysis modalities, offering opportunities for few-cell and single-cell analysis of adherent eukaryotic cells. We demonstrate that light microscopy can be directly combined with single-cell LC-MS for metabolite detection and single-cell RPPA for protein quantification. However, the correlative analysis for all three information channels (light microscopy, LC-MS, RPPA) only works reliably for the few-cell analysis. For quantitative measurements using single individual cells, the transfer carrier must be further improved for better sample localization and protein retention.

We foresee this methodology as a versatile tool to study a mixture of adherent eukaryotic systems. A typical example would be an ensemble of different interacting cell types, *e.g.*, a model system to study the prion-like spreading of (fluorescently labeled) amyloids (Fig. 1d and S3†) from diseased to healthy cells. The precise preselection of the cell by its visual appearance (fluorescence signal of amyloid particles) is crucial. Otherwise, the measurements are obscured by unaffected cells and different cell types. Finally, the complex interplay of the amyloids with other proteins and lipids must be characterized in a multi-omic approach, as presented here.

## Data availability

Data for this article and ESI,† including extracted mass spectrometry data or mzML files and the data analysis scripts (Jupyter Notebooks with Python), are available at Zenodo at https://doi.org/10.5281/zenodo.11637652.

## Author contributions

L. R. and S. A. performed the cell experiments in the cryoWriter setup and the RPPA analysis. C. B. performed the mass spectrometry measurements. R. S. and A. F. were responsible for cell culturing, and R. S. provided expertise in immunolabeling. G. D. performed the first RPPA experiments. G. S. coordinated the mass spectrometry measurements. T. B. coordinated the project. L. R., S. A., C. B., and T. B. wrote the manuscript. All authors commented on and discussed the work.

## Conflicts of interest

The cryoWriter is an element of the PCT/EP2015/065398 patent application. Furthermore, TB is a co-founder and board member of cryoWrite AG, developing instruments for electron microscopy.

## Supplementary Material

LC-024-D4LC00269E-s001

LC-024-D4LC00269E-s002
