# Supplementary material for: Single and few cell analysis for correlative light microscopy, metabolomics, and targeted proteomics
Source: Lab Chip. 2024 Aug 8;24(18):4321–32. doi: 10.1039/d4lc00269e (PMC11318241; doi:10.1039/d4lc00269e)
Supplement: LC-024-D4LC00269E-s002 [file LC-024-D4LC00269E-s002.pdf]

Supplementary Information (SI) for Lab on a Chip.
This journal is © The Royal Society of Chemistry 2024

Supplementary material

Single and few cell analysis for correlative light microscopy, metabolomics, and targeted proteomics

Luca Rima,[*,a] Christian Berchtold,[*,b,d] Stefan Arnold,[*,a,c] Andri Fränkl,[a] Rosmarie Sütterlin,[a] Gregor Dernick,[e] Götz Schlotterbeck,[b] and Thomas Braun[a]

[a] *Biozentrum, University of Basel, Spitalstrasse 41, Basel, Switzerland.*
[b] *FHNW Fachhochschule Nordwestschweiz, Hochschule für Life Sciences E-mail: christian.berchtold@fhnw.ch, goetz.schlotterbeck@fhnw.ch*
[c] *Current address: Sensirion AG, Laubisruetistrasse 50, 8712 Stäfa*
[d] *Current address: Zentrum für Forensische Haaranalytik, Universität Zürich, Institut für Rechtsmedizin, Kurvenstrasse 17, 8006 Zürich, Switzerland*
[e] *F. Hoffmann-La Roche Ltd*
[*] *Equally contributed.*

A Dispensing precision

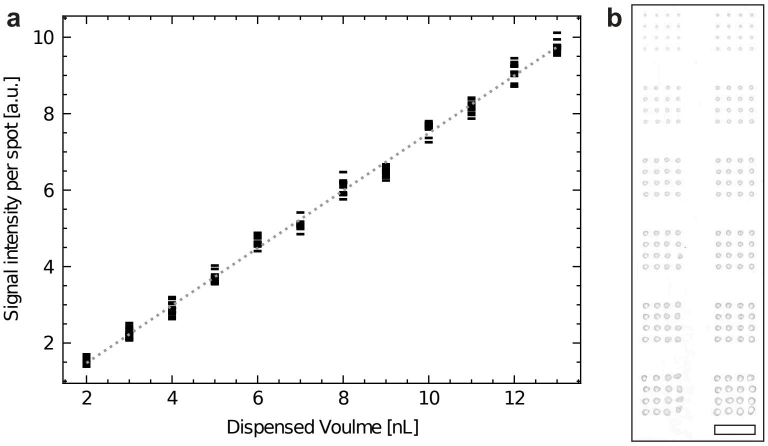

Fig. SI-1 a) Multiple arrays of 4x4 spots of 10 nM sulforhodamine b solution were spotted on a clean glass slide. The spotted volume was increased by 1 nL with every new array (from 2 nl to 13 nl). The spotted volume was plotted against signal intensity. **b)** An inverted image of the scanned slide shows the increasing spot size and total signal intensity with increasing spot volume. Scale bar is 5 mm.

B Single cell lysis movie

Fig. SI-2 Typical single cell lysis movie (file: CellLysis_Trim.mp4). HEK cells were grown on functionalized ITO-coated microscopy glass slides and targeted by Pt-coated, conducting microcapillary. Cell lysis is performed by the combined forces of electroporation and friction force by the suction of the sample volume.

C Single cell lysis: effects on surrounding cells

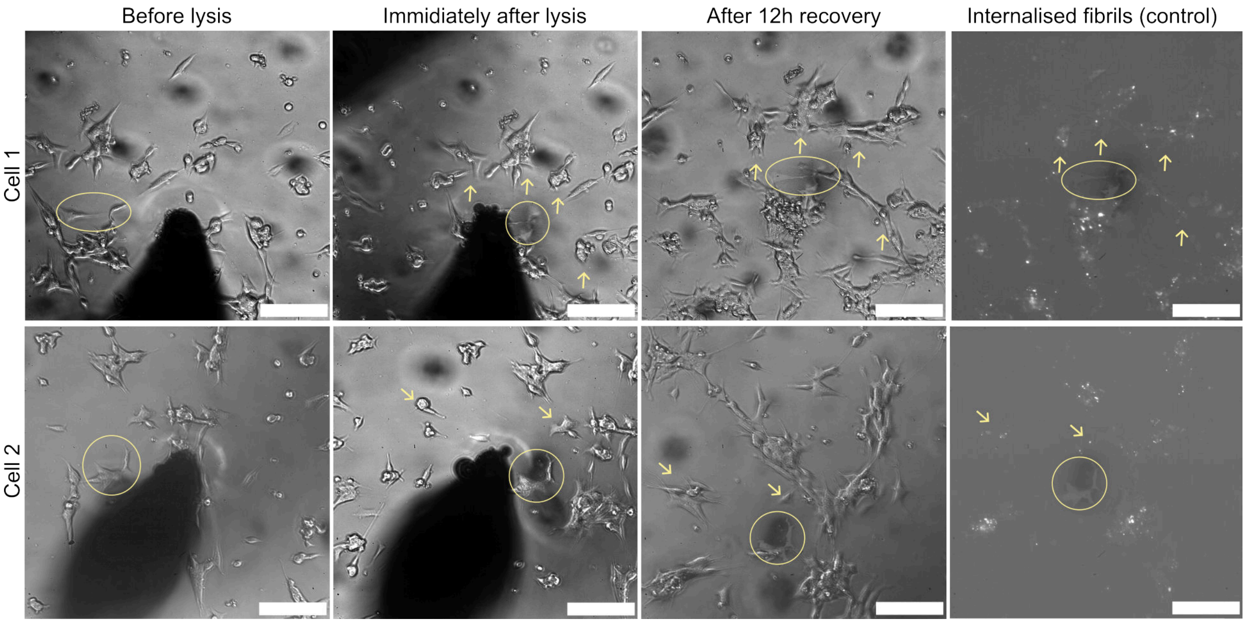

Fig. SI-3 Monitoring effects of the single-cell electroporation on neighboring cells. An individual cell was electroporated (yellow circle) and aspirated into the microcapillary. Afterward, the cell culture was placed in the incubator for an additional $12\,\mathrm{h}$ cell growth (recovery), and the same place was imaged again by light microscopy. The results show that (i) cells in proximity ($>50\,\mu\mathrm{m}$ distance to the lysis center) are still able to divide and proliferate (yellow arrows), and (ii) the fluorescence-labeled amyloid particles remain in the cells.

D Control software

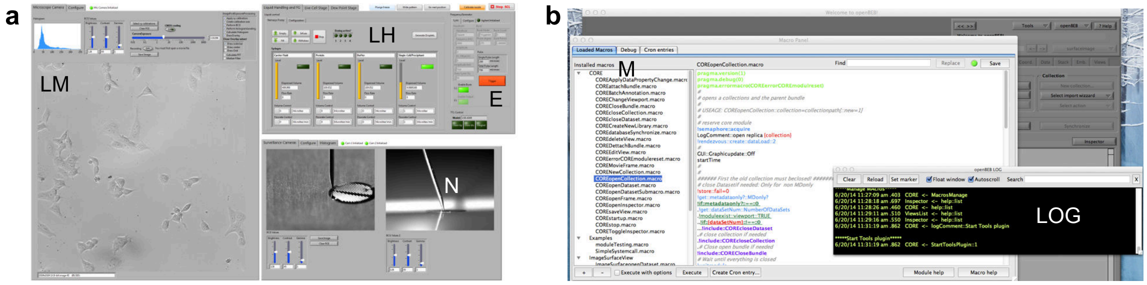

Fig. SI-4 Screenshots of the graphical user interface (GUI) of the control software (a) and the macro and logging system of openBEB[1] (b). **a)** GUI of the control software used for single-cell lysis. The microscopy view (LM) is shown with HEK cells, the panel for the liquid handling (LH), and the observation camera with the nozzle (N). **b)** The instrument can be automatized by an extensive, domain-specific macro language by openBEB's macro subsystem; here, the integrated macro editor (M) is shown, additionally providing an extensive logging facility (LOG).

E Additional RPPA data and comparison with bach cell lysate

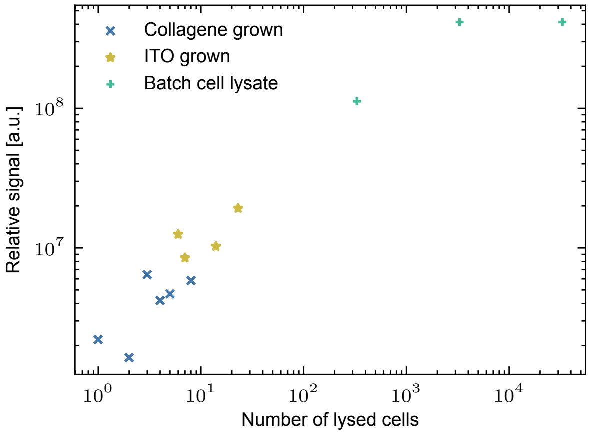

Fig. SI-5 RPPA analysis of HEK cells grown on different substrates (collagen functionalized, directly on ITO) and comparison with batch cell lysate. Individual cells were lysed and spotted on the NC-coated glass slides. Actin was detected by the primary antibody, and the fluorescently labeled secondary antibody performed quantification. Note the large dynamic scale of the signal and the oversaturation at the highest cell counts in the batch lysate.

F Handover substrates

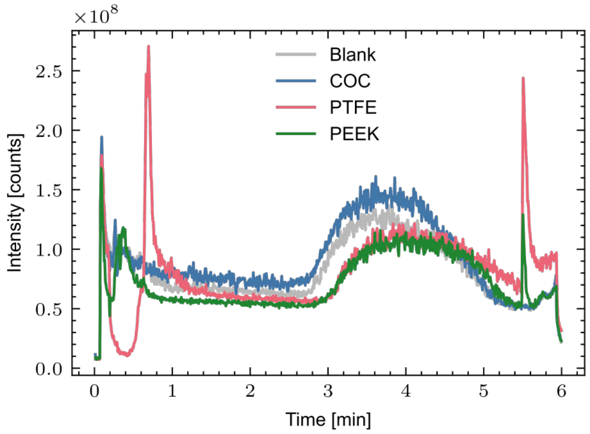

Fig. SI-6 The slides must resist the extraction solvents used for LC-MS, mechanically withstand the forces induced by the extraction head, enable proper sealing with the extraction interface, and retain proteins on the slide for the subsequent analysis by RPPA. Unfortunately, classical NC-coated slides routinely used for RPPA analysis[2] were too fragile for the extraction process needed for LC-MS analysis. Tests with the unmodified TLC device were performed using COC, PEEK, and PTFE slides. All materials were washed with water, acetonitrile, and isopropanol. No significant contamination was found compared to a blank test without a slide. Sealing on all substrates was sufficient up to $400\,\mathrm{bar}$. The tests were performed without an LC column, with a mass range 50-2000 m/z positive and negative mode. Full scan orbit-trap (resolution 7500). Blank = no extraction over the slide. Gradient $100\,\%$ H2O, $0.1\,\%$ formic acid, to $100\,\%$ acetonitrile/isopropanol, $0.1\,\%$ formic acid. Ultimately, COC was selected for experiments employing non-functionalized slides, primarily owing to its availability and material transparency. This choice also facilitated the visual identification of sample spots by eye or light microscopy.

G COC slides

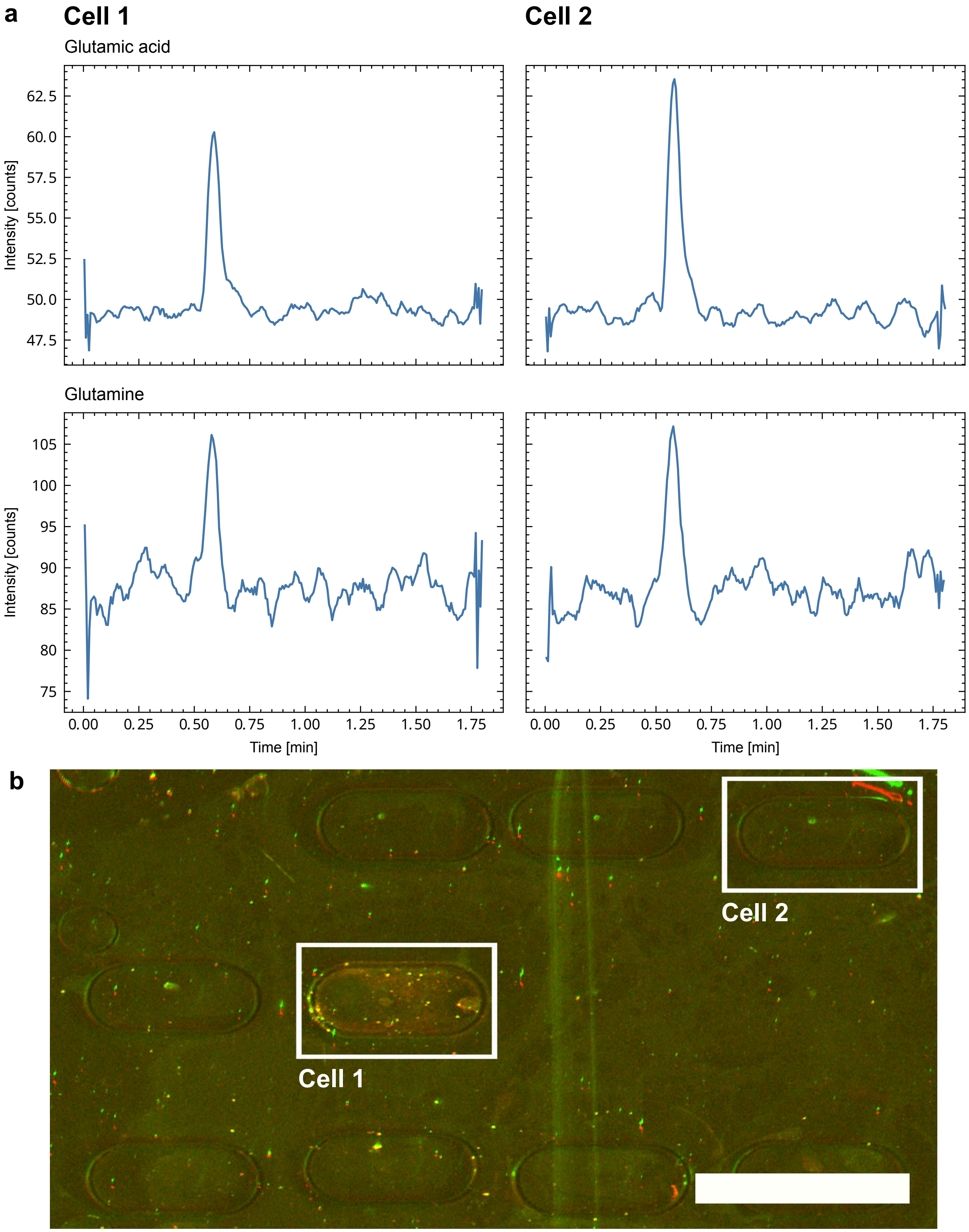

Fig. SI-7 Using COC slides, HPLC-MS analysis was feasible, as can be seen in panel **(a)** for glutamine and glutamic acid. The immunolabeling yielded no distinct results. The white frames in panel **(b)** indicate the areas of measurement. Scale bar: 5 mm.

H 3D printed polypropylen cap

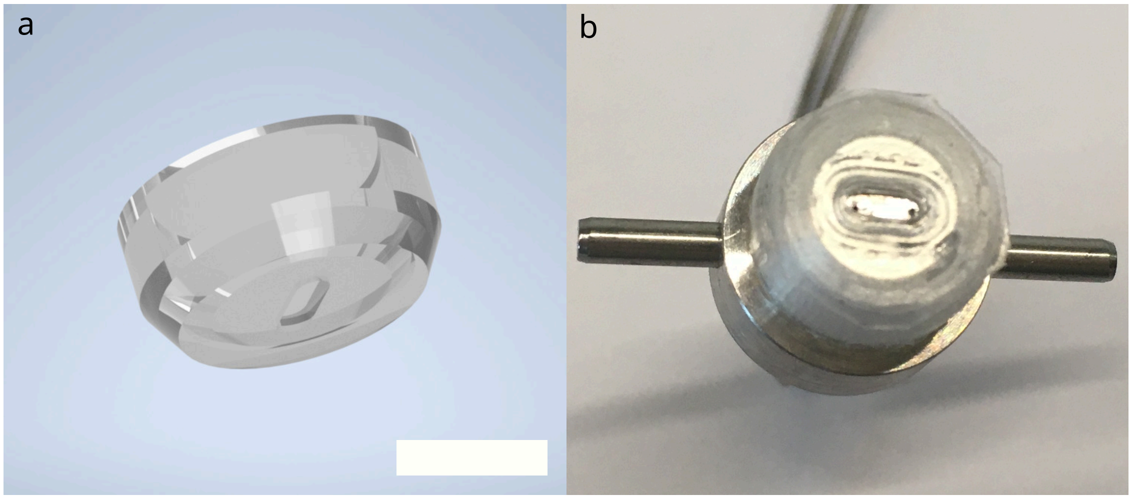

Fig. SI-8 (a) Rendered image of the 3D printed polypropylen cap for sealing. Scale bar: 5 mm. **(b)** Picture of the cap mounted on the extraction head of the TLC-MS Interface 2.

I Mass spectra

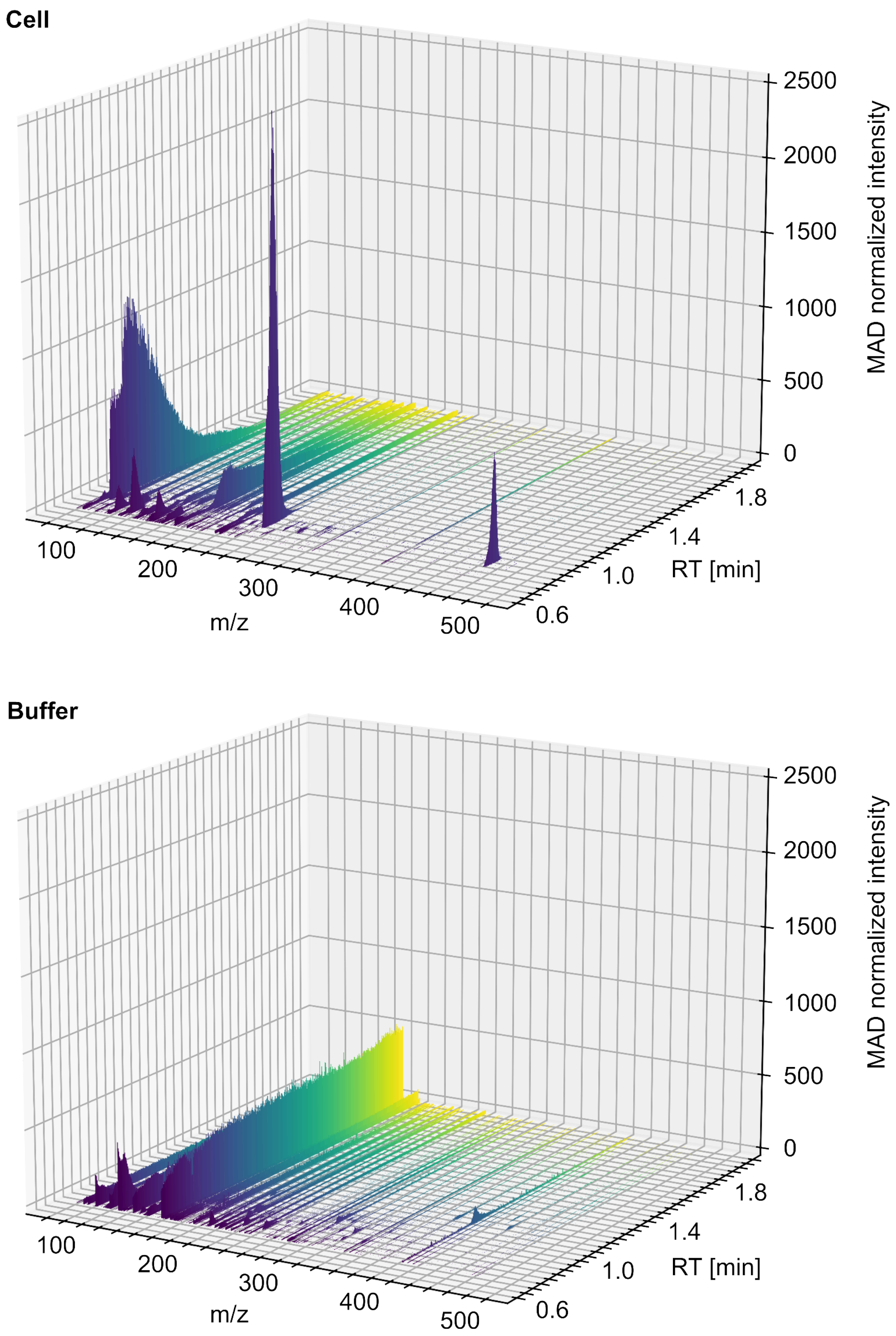

Fig. SI-9 Extracted mass spectra. Mass range 0 to 500 with the retention time (RT) $0.5\,\mathrm{min}$ to $2\,\mathrm{min}$ is shown. Nine lysed cells are compared with a buffer surrounding the cells of the same volume. Centroid mode intensities were normalized with the Median Absolute deviation (MAD). The color coding is used for better visibility. Different metabolites can be seen, which are also present in the buffer. However, there is a clear difference in intensity and number of signals. Note that the cells dilute the buffer during cell lysis and uptake.

Notes and references

1 C. Ramakrishnan, A. Bieri, N. Sauter, S. Roizard, P. Ringler, S. A. Müller, K. N. Goldie, K. Enimanev, H. Stahlberg, B. Rinn and T. Braun, *BMC Bioinformatics*, 2014, **15**, 84.

2 G. Dernick, S. Obermüller, C. Mangold, C. Magg, H. Matile, O. Gutmann, E. v. d. Mark, C. Handschin, C. Maugeais and E. J. Niesor, *Journal of Lipid Research*, 2011, **52**, 2323–2331.
